# Glutathione Metabolism Is a Regulator of the Acute Inflammatory Response of Monocytes to (1→3)-β-D-Glucan

**DOI:** 10.3389/fimmu.2021.694152

**Published:** 2021-11-11

**Authors:** Rayoun Ramendra, Mathieu Mancini, Jose-Mauricio Ayala, Lin Tze Tung, Stephane Isnard, John Lin, Jean-Pierre Routy, Anastasia Nijnik, David Langlais

**Affiliations:** ^1^ Department of Microbiology and Immunology, McGill University, Montreal, QC, Canada; ^2^ McGill University Research Centre on Complex Traits, Montreal, QC, Canada; ^3^ McGill University Genome Centre, Montreal, QC, Canada; ^4^ Department of Human Genetics, McGill University, Montreal, QC, Canada; ^5^ Department of Physiology, McGill University, Montreal, QC, Canada; ^6^ Chronic Viral Illness Service, McGill University Health Centre, Montreal, QC, Canada

**Keywords:** B-glucan, glutathione, immunometabolism, innate immunity, host response

## Abstract

(1→3)-β-D-Glucan (BDG) represents a potent pathogen-associated molecular pattern (PAMP) in triggering the host response to fungal and some bacterial infections. Monocytes play a key role in recognizing BDG and governing the acute host response to infections. However, the mechanisms regulating monocyte’s acute response to BDG are poorly understood. We sought to investigate the response of monocytes to BDG at the epigenetic, transcriptomic, and molecular levels. Response of human monocytes to 1, 4, and 24 hours of BDG exposure was investigated using RNA-seq, ATAC-seq, H3K27ac and H3K4me1 ChIP-seq. We show that pathways including glutathione metabolism, pentose phosphate pathway, and citric acid cycle were upregulated at the epigenetic and transcriptomic levels in response to BDG exposure. Strikingly, unlike bacterial lipopolysaccharides, BDG induced intracellular glutathione synthesis. BDG exposure also induced NADP synthesis, increased NADPH/NADP ratio, and increased expression of genes involved in the pentose phosphate pathway in a GSH-dependent manner. By inhibiting GSH synthesis with L-buthionine sulfoximine (BSO) before BDG exposure we show that the GSH pathway promotes cell survival and regulates monocyte’s effector functions including NO production, phagocytosis, and cytokine production. In summary, our work demonstrates that BDG induces glutathione synthesis and metabolism in monocytes, which is a major promoter of the acute functional response of monocytes to infections.

## Introduction

(1→3)-β-D-Glucan (BDG) represents one of the most abundant components of the fungal and some bacterial cell walls and is a potent pathogen-associated molecular pattern (PAMP) in triggering the host response to infections (1). For example, invasive fungal infections (IFIs) represent a rising cause of human disease with increased use of immunosuppressive therapies, broad-spectrum antibiotics, and invasive medical devices. Such infections are a common complication for a wide spectrum of immunocompromised patients including people living with HIV, cancer, solid organ transplant, systemic lupus erythematosus, and other predisposing conditions like diabetes and pregnancy (2). BDG assays are now widely used as diagnostic tools for identifying mycosis in clinical settings (3–5). Recent studies have shown that circulating BDG levels are linked to immune activation and inflammation in people living with chronic conditions that induce epithelial gut damage and subsequent microbial translocation such as CMV and HIV infections (6–9). A growing body of literature has highlighted the capacity of BDG to induce long-term epigenetic reprogramming in innate immune cells, termed trained immunity, which leads to an adjusted response to a subsequent challenge and to some metabolic changes, including a switch from oxidative phosphorylation to aerobic glycolysis (10, 11). Similarly, BDG has been shown to be able to reverse LPS-induced tolerization in monocytes/macrophages and confer protection against infectious diseases including leishmaniasis and tuberculosis (12–14).

Circulating monocytes serve as an integral part of the host response to infections by detecting PAMPs, phagocytosing/presenting antigens, and producing pro-inflammatory cytokines/chemokines to help recruit a larger wave of diverse leukocytes to the site of infection. In humans, monocytes can recognize BDG using the C-type lectin receptor Dectin-1 and complement receptor 3 (CD11b/CD18 heterodimer) (15). Dectin-1 deficiency has been shown to be associated with increased susceptibility and significantly impaired immune response to *Candidiasis* by monocytes/macrophages in both mice and humans (16, 17). While the acute recognition and response of monocytes to BDG is widely recognized as a critical component of the host’s response to various infections, the mechanistic pathways implicated in this response remain to be fully understood.

Herein, we exploited previously published epigenetic and transcriptomic data on monocytes exposed to BDG (12) to unveil novel components of the host response to BDG. We confirmed that BDG induces increased expression of genes involved in glucose metabolism, including pentose phosphate and cholesterol metabolism. We expand such knowledge by revealing for the first time that BDG stimulation induces epigenetic and transcriptomic changes in monocytes associated with increased glutathione synthesis and metabolism. Interestingly, intracellular glutathione levels were crucial in the regulation of several of the monocyte’s antifungal functions including resilience to oxidative stress, immunometabolism, nitric oxide production, phagocytosis, and cytokine production.

## Materials and Methods

### Monocytes from Healthy Donors

All primary cells for *in vitro* experiments were obtained from healthy donors who gave written informed consent (Chronic Viral Illness Service, at McGill University Health Centre (MUHC) Montreal, QC, Canada) and approved by the REB (2019–5170) of MUHC. Peripheral blood mononuclear cells (PBMC) were isolated by leukapheresis and stored in liquid nitrogen. Cells were rapidly thawed and rested for 1 hour at 37°C. Monocytes were purified from PBMC using a negative selection Human Monocyte Isolation Kit (StemCell Easy Sep). Successful isolation of monocytes was confirmed with flow cytometry (BD Fortessa) using VivaFix Viability Assay (BioRad), a cocktail of antibodies from BioLegend: anti-CD3 PE (300456), anti-CD14 BV650 (301835), anti-CD19 PerCP-Cy5.5 (302229), and anti-CD56 APC (318309). The gating strategy for the live single-cell CD3- CD19- CD56- CD14+ population is presented in **Supplementary Figure 1**. The purity of the isolated monocytes can be found in (**Supplementary Table 1**).

Our monocyte isolation protocol is similar to *Novakovic* et al. where they have enriched monocytes from healthy volunteers to generate the functional genomics datasets reanalyzed herein (12). Briefly, they isolated peripheral blood mononuclear cells (PBMC) using centrifugation in Ficoll-Paque (GE Healthcare), followed by an additional Percoll gradient to remove T cells. Monocytes were then purified using negative selection in an LD column magnet separator (Miltenyi Biotech) and monocytes purity was assessed using flow cytometry.

### Cell Culture

Monocytes were cultured in complete media consisting of Dulbecco’s Modified Eagle Medium containing high glucose and sodium pyruvate (ThermoFisher Scientific) with 10% heat-inactivated fetal bovine serum (Wisent BioProducts) and 100U/mL penicillin/streptomycin (Corning). NGS datasets produced by *Novakovic* et al. were produced using 5ug/mL of (1→3)-β-D-glucan from heat killed *Candida albicans* for 24 hours, as previously described (12). Cells in our *in vitro* experiments were stimulated with 5μg/mL of (1→3)-B-D-glucan from *Alcaligenes faecalis* (Sigma-Aldrich), 100ng/mL of lipopolysaccharide (LPS) from *Escherichia coli* O127:B8 (Sigma-Aldrich), 120μM L-buthionine sulfoximine (Sigma-Aldrich), and/or 1mg/mL of reduced glutathione (Sigma-Aldrich) for 24 hours unless otherwise stated in figure legends.

### ATAC-Seq Peak Finding Analysis

**Figure 1:**
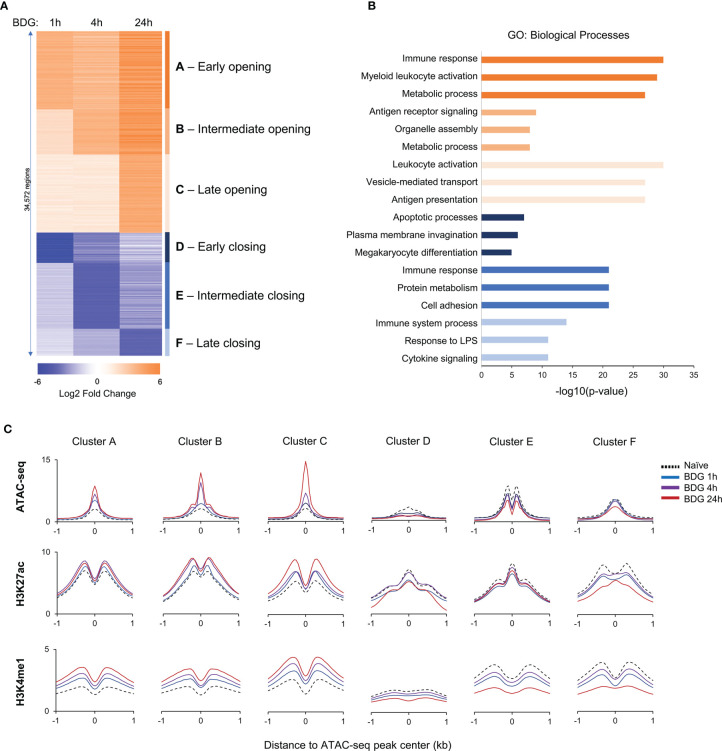
Changes in the epigenetic landscape of monocytes after BDG exposure. **(A)** Heatmap of differentially accessible regions (DARs) after BDG exposure identified using ATAC-seq; fold change ≥ |2|. Regions were clustered (groups A-F) based on increase or decrease of chromatin accessibility after 1h, 4h, and 24h of BDG exposure. **(B)** Gene ontology enrichment analysis for each DAR group defined in panel A; the nearest gene to each DAR with a maximum distance of 500kb were used for the analysis. The top three categories are shown; see **Supplementary Table 2B** for complete list. **(C)** Density profiles of ATAC-seq, H3K27ac and H3K4me1 ChIP-seq at the DAR clusters. Signal is measured in 50bp bins ( ± 1kb) centered on the ATAC-seq peaks.

The following published ATAC-seq datasets were analyzed: untreated, BDG 1h, BDG 4h, and BDG 24h stimulated monocytes (GEO: GSE85246) (12). Sequence reads were downloaded using fastq-dump from the SRA Toolkit with the setting –split-files and mapped to the human hg19 reference genome assembly with Bowtie 2.4.0 (18). The files containing mapped reads were converted from SAM to BAM format using samtools 0.1.18 (19) and then Tag directories were generated for further analysis using the Homer toolkit (20). To identify accessible genome regions, we processed the mapped reads with MACS 1.4.1 (21) with a p-value cutoff of 1E-8. Significant peaks that were less than 100bp apart were merged using MergePeaks and subsequently annotated using AnnotatePeaks from the Homer toolkit (20). 55,971 regions were identified to have accessible chromatin in one or more of the four conditions and read densities were queried at each location (+/- 100bp of the peak center) in all four conditions using the AnnotatePeaks function (20). 34,572 differentially accessible regions (DARs) between conditions were then identified using a fold-change threshold of ≥ |2| when compared to the untreated condition (**Supplementary Table 2A**). These regions were clustered based on their dynamics relative to the untreated condition and the heatmap (**Figure 1A**) was generated using Java TreeView (22). Sequence read density profiles (bigwigs) were generated from BAM files using bamCoverage from the deepTools 3.1.1 toolkit (23).

### Transcription Factor Binding Motifs Analyses

Known and *de novo* transcription factor binding motifs were identified in the ATAC-seq DAR clusters using the findMotifsGenome tool from Homer using the settings: -size 200 -mask. The motifs found to be enriched in one of the clusters were then queried to find their relative frequency within each cluster *vs* randomly generated sets of background sequences using the Homer toolkit (20). Potential transcriptional regulatory networks were identified by querying the list of dysregulated genes from RNA-seq against a database of TF cistromes using TRRUST (24).

### ATAC-Seq Gene Ontology Analyses

Genomic coordinates for each DAR cluster ATAC-seq peaks were submitted to the GREAT 2.0.2 tool to determine if specific biological functions were enriched with differentially accessible regions at different times (25). We used 500kb as the maximum absolute distance to the nearest transcriptional start site and q-values less than 1E-10 as statistically significant. Representative Gene Ontology (GO) biological process categories were selected to remove redundancy and reported alongside the ATAC-seq DAR clusters; the full list of enriched GO categories can be found in (**Supplementary Table 2B**).

### ChIP-Seq Peak Finding Analyses

H3K27ac and H3K4me1 ChIP-seq datasets for untreated, BDG 1h, BDG 4h, and BDG 24h stimulated monocytes were downloaded using fastq-dump from the SRA toolkit (GEO: GSE85246) (12). Sequence reads were aligned to the human hg19 reference genome using Bowtie 2.4.0 (18). The files containing mapped reads were converted from SAM to BAM format using samtools 0.1.18 (19) and formatted into Tag directories for further analysis using the Homer toolkit (20). Bigwigs were generated from BAM files using bamCoverage from the deepTools 3.1.1 toolkit (23).

### ATAC-Seq and ChIP-Seq Density Profiles

AnnotatePeaks from the Homer toolkit (20) was used to query the read density at the genomic coordinates of the DAR clusters in the ATAC-seq, H3K27ac ChIP-seq, and H3K4me1 ChIP-seq datasets ±1kb centered around the ATAC-seq peak. Data was visualized in Microsoft Excel 2010 comparing ATAC-seq and ChIP-seq signals in each cluster after varying durations of BDG exposure.

### RNA-Seq Analyses

Biological duplicates of RNA-seq on human monocytes untreated or stimulated with BDG for 1 hour, 4 hours, or 24 hours were downloaded using the SRA toolkit using –fastq-dump (GEO: GSE85246). The quality of sequence reads was confirmed using FastQC (Babraham Bioinformatics) and low-quality reads and bases were trimmed using Trimmomatic v.0.3 (26). Reads were mapped to the human hg19 assembly using HISAT2 (27). Resulting SAM files were then converted to BAM format using samtools 0.1.18 (19). Gene expression was quantified by counting the number of uniquely mapped reads to exons with featureCounts (28). Normalization and differential gene expression analysis was conducted using the edgeR Bioconductor package (29). Genes with at least three counts per million reads in at least two samples were retained for pairwise differential gene expression analyses comparing BDG exposed and unexposed monocytes. Genes with differential expression > |2| and FDR (p-value corrected using Benjamini-Hochberg method) <1x10^-3^ were considered significant and clustered based on differential expression compared to untreated monocytes (**Supplementary Table 3A**). Clusters were visualized using Java TreeView (22). Bigwigs were made using genomeCoverageBed from bedtools 2.17.0 and wigToBigWig from the UCSC toolkit after scaling per 10 million reads mapping onto exons (30, 31). Gene ontology enrichment analyses was conducted on differentially expressed gene clusters by submitting gene sets to EnrichR (**Supplementary Table 3B**) (32).

### RNA Extraction, cDNA Synthesis, and Real-Time qPCR

RNA was extracted using EasyPure RNA Extraction Kit (Transgen Biotech) and cDNA was synthesized using the TransScript One-Step gDNA Removal and cDNA Synthesis kit (Transgen Biotech). RT-qPCR was performed with the Luna Universal qPCR Master Mix (New England BioLabs) using the primers listed in (**Supplementary Table 4**). Data was analyzed using CFX Maestro Software for Real-Time PCR (BioRad) and normalized to *ACTB* gene expression.

### Monocyte Function

Cell viability was assessed using Trypan blue and counted using a hemocytometer. Intracellular reduced and oxidized glutathione levels were measured and normalized per 1 million cells as previously described (33). NADP and NADPH levels were measured using an NADP/NADPH Assay (Abcam) and normalized by cell count. Nitric oxide production was assessed by a Griess assay on the culture supernatant (34). Phagocytic capacity of monocytes was assessed by exposing the monocytes to the pHrodo Green *E. coli* BioParticles Conjugate for Phagocytosis (ThermoFisher Scientific) for 30 minutes and the endpoint signal was measured using a spectrophotometer at OD 495nm. IL-6, IL-8, and IL10 levels were measured in culture supernatant using Quantikine Human ELISA kits (R&D Systems).

## Results

### Epigenetic Landscape of Monocytes Exposed to BDG

To investigate the functional changes driven by acute exposure of human monocytes to BDG, we have reanalyzed available functional genomics datasets (12). We first assessed acute changes in chromatin accessibility before and after exposure to BDG for 1, 4, and 24 hours. As expected, BDG induced significant changes in chromatin accessibility with a total of 34,572 differentially accessible regions (DARs) before and after different durations of BDG exposure. We grouped these regions based on (1) early, (2) intermediate, and (3) late chromatin opening, as well as (4) early, (5) intermediate, and (6) late chromatin closing (**Figure 1A** and **Supplementary Table 2A**). 21,870 regions had increased accessibility after BDG exposure (groups A-C) whereas 12,702 regions had decreased accessibility (groups D-F). GO analysis was then conducted to identify biological processes associated with these groups (**Figure 1B** and **Supplementary Table 2B**). DAR groups associated with increased chromatin accessibility (A-C) were proximal to genes involved in myeloid leukocyte activation, metabolic processes, antigen receptor signaling, organelle assembly, vesicle-mediated transport, and antigen presentation. Regions with decreased accessibility were associated with apoptotic processes, plasma membrane invagination, protein metabolism, cell adhesion, response to LPS, and cytokine signaling.

We next evaluated the levels of H3K27ac, a mark of active cis-regulatory regions, and H3K4me1, mark of active and primed enhancers, in monocytes before and after 1, 4, and 24 hours of BDG exposure. Groups A-C were associated with significantly increased H3K27ac and H3K4me1 signals around the ATAC-seq peak and groups D-F were associated with significantly decreased signals (**Figure 1C** and **Supplementary Figure 2**). Globally, the timing of these changes in histone modifications matched changes observed in ATAC-seq signal.

### Transcriptomic Changes in Monocytes Exposed to BDG

**Figure 2:**
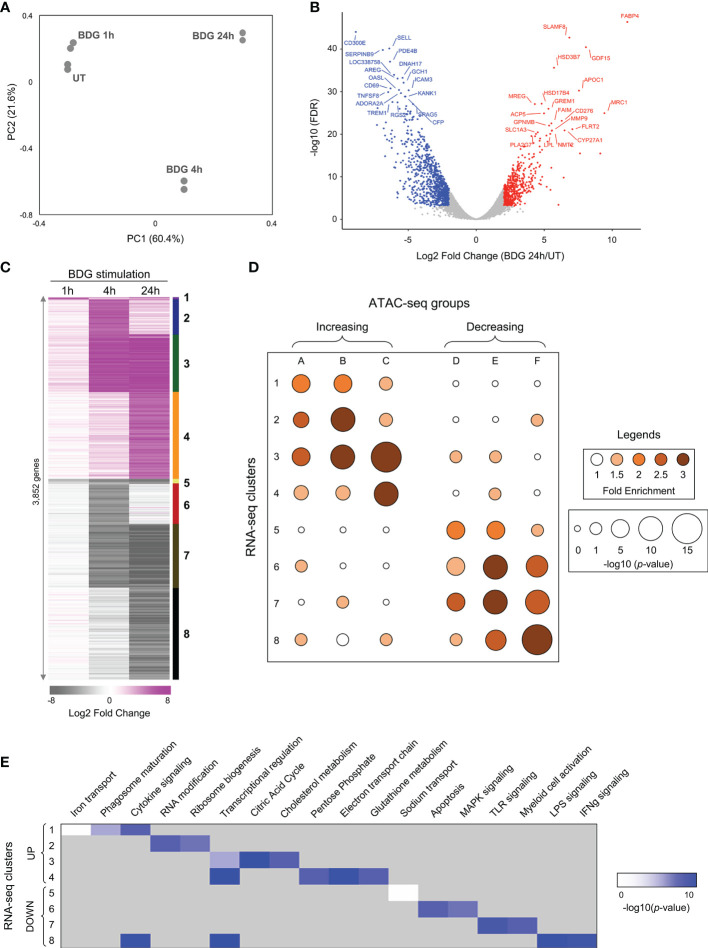
Differential gene expression of monocytes after exposure to BDG. **(A)** Principal component analysis of RNA-seq data from untreated monocytes (UT) or monocytes exposed to BDG for 1h, 4h, and 24h. **(B)** Volcano plot of differentially expressed genes after 24h of BDG exposure compared to resting monocytes. False discovery rates (FDR) are calculated using Fisher’s exact test with correction using the Benjamini and Hochberg method. **(C)** Heatmap identifying groups of genes that are differentially expressed after 1, 4, and 24 hours of BDG exposure. Genes are grouped (1–8) based on a cutoff of fold-change > 2 (upregulated) or < 0.5 (downregulated) and FDR < 0.001. **(D)** Bubble histogram showing the association between groups of differentially accessible regions and clusters of differentially expressed genes after BDG exposure. The color gradient reflects the ratio in the number of regions in each cluster associated with differentially expressed gene groups compared to control sets of randomly selected genes. Bubble size indicates the strength of the –log10 Fisher’s exact test p-value for the association between DARs and differentially expressed genes compared to randomly selected control genes. **(E)** GO category enrichment analysis for groups of differentially expressed genes (white to blue gradient represents degree of enrichment, grey – not enriched).

To further characterize the acute response of monocytes to BDG we assessed transcriptomic changes using RNA-seq. Principal component analysis clearly demonstrates that duration of BDG exposure (PC1) has a major effect on monocyte gene expression (**Figure 2A**). Moreover, monocytes exposed to BDG for 4 hours have a different transcriptomic program compared to resting monocytes and monocytes exposed to BDG for 1 hour and 24 hours (PC2). After 24 hours of BDG exposure, we identified 3,852 genes with significantly dysregulated expression compared to resting monocytes (FC≥|2| and FDR ≤ 0.001) (**Figure 2B**). Genes that were significantly dysregulated after BDG exposure were clustered as (1) early and stable upregulation, (2) transient upregulation at 4 hours, (3) intermediate and stable upregulation, (4) late upregulation, (5) early and stable downregulation, (6) transient downregulation at 4 hours, (7) intermediate and stable downregulation, and (8) late downregulation (**Figure 2C** and **Supplementary Table 3A**). There is a high degree of correlation between the ATAC-seq groups and RNA-seq clusters in both direction and timing of differential chromatin accessibility and gene expression (**Figure 2D**), showing that the BDG-induced changes in chromatin accessibility are associated with transcriptional changes of proximal genes. Gene ontology enrichment analyses were performed to identify biological processes enriched in these RNA-seq clusters (**Figure 2E** and **Supplementary Table 3B**). As previously described, the classical proinflammatory categories (i.e., TLR, MAPK, LPS, and IFNγ signaling) were enriched in the downregulated clusters (12, 35). Genes linked to cytokine signaling and transcriptional regulation were enriched in both up and down regulated gene sets. Interestingly, the categories enriched in the upregulated clusters are related to phagosome maturation, RNA and translation, but importantly multiple metabolism pathways including citric acid cycle, cholesterol metabolism, pentose phosphate pathway, electron transport chain, and glutathione metabolism.

### Transcriptional Regulation of Monocytes Exposed to BDG

**Figure 3:**
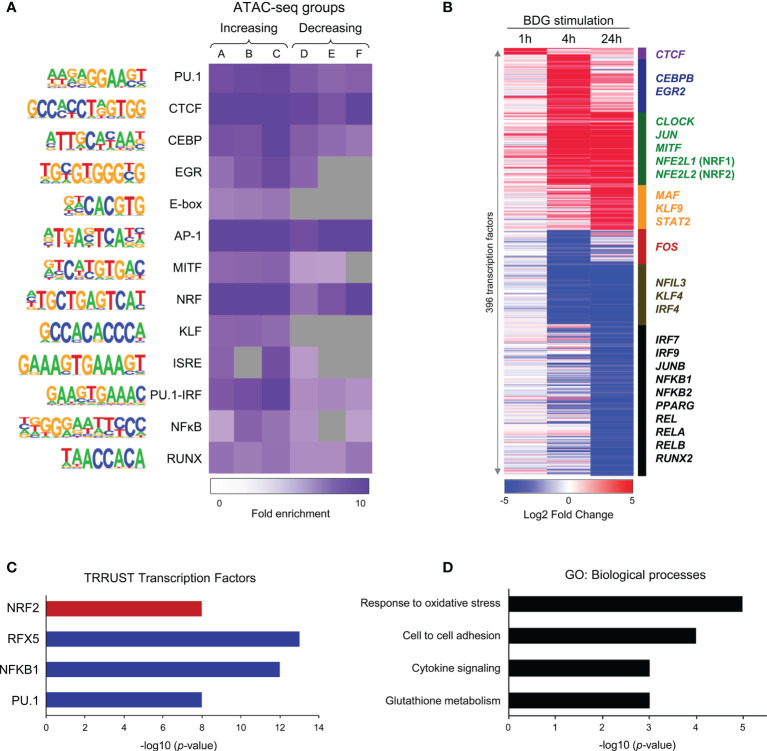
Changes in motif enrichment and transcription factor expression in monocytes after BDG exposure. **(A)** Enriched motifs using Homer *de novo* motif analysis on the DAR clusters identified in **Figure 1A**. Color gradient indicates ratio of motif enrichment amongst regions in that cluster compared to randomly selected regions across the human genome with similar GC-content. Grey boxes indicate fold enrichment less than 1. **(B)** Heatmap of 342 differentially expressed transcription factors (TFs) after BDG exposure in the groups defined in **Figure 2C**. **(C)** TRRUST query of genes significantly dysregulated by 24h BDG exposure *vs* random sets of genes against a database of all known cistromes. Red represents cistromes enriched in genes significantly upregulated by BDG exposure, blue represents cistromes enriched amongst genes significantly downregulated by BDG exposure (p < 10^-7^). **(D)** Biological process gene ontology enrichment analysis of genes significantly upregulated by BDG exposure and enriched in the NRF2 cistrome (p < 0.01).

As the “transcriptional regulation” GO category was enriched in both up and downregulated genes clusters (**Figure 2E**), we further used the ATAC-seq and RNA-seq datasets to gain insight in the transcriptional regulation of the monocytes’ response to BDG. Using Homer (20), we search for enrichment of various transcription factors (TFs) binding motifs in the ATAC-seq groups (**Figure 3A**). As expected, the motif of pioneer TFs (PU.1, CEBP, and AP-1) as well as architectural TF CTCF were enriched in all clusters. EGR and MITF binding motifs, previously shown to be enriched after BDG exposure in this dataset (12), were enriched in all ATAC-seq groups with increased DARs. The E-box and KLF motifs, were enriched in groups linked to increased but not decreased chromatin accessibility. The NRF motif, associated with the antioxidant response in myeloid cells (36), was enriched in all the groups with the highest levels of enrichment in clusters with increased chromatin accessibility (A-C) and cluster F. We then extracted the differentially expressed TF expression using the same clustering from **Figure 2C** (**Figure 3B** and **Supplementary Table 3C**). CTCF, CEBP, EGR, E-box, AP-1, MITF, and NRF motifs had enrichment in the ATAC-seq groups and the expression of genes encoding TF of these families were significantly upregulated (i.e. *CTCF, CEBPB, EGR2, CLOCK, JUN, MITF and NFE2L1-2*, respectively), while IRF, NFκB and RUNX family members were downregulated. Using TRRUST (24) we queried differentially up and down regulated genes in response to BDG exposure against a database of TF cistromes based on ChIP-chip and ChIP-seq datasets (**Figure 3C** and **Supplementary Table 5A**). Interestingly, the NRF2 cistrome was significantly enriched amongst the genes upregulated after BDG exposure. Gene ontology of genes in the NRF2 cistrome that were also upregulated by BDG exposure were enriched for the biological processes: response to oxidative stress, cell-cell adhesion, cytokine signaling, and glutathione metabolism (**Figure 3D** and **Supplementary Table 5B**).

### BDG Induces Glutathione Synthesis and Metabolism in Human Monocytes

**Figure 4:**
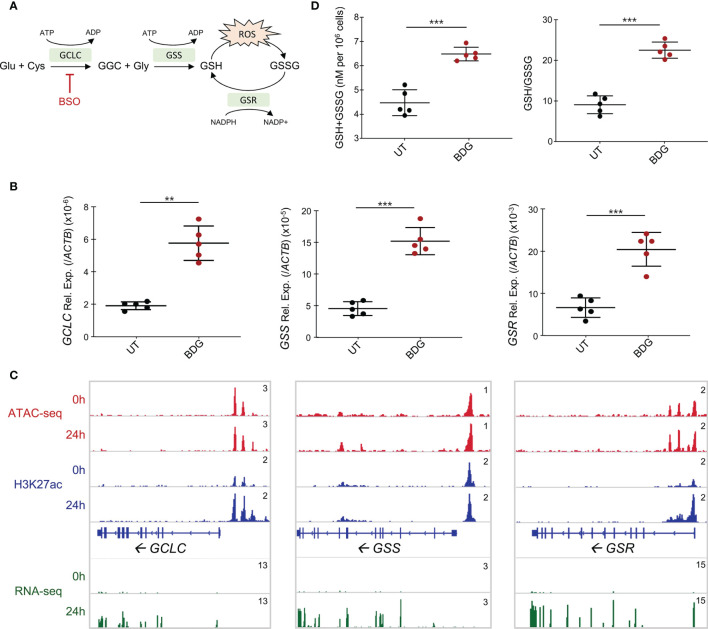
Glutathione synthesis and metabolism are induced by BDG stimulation. **(A)** Schema of glutathione metabolic pathway with genes that are significantly upregulated from the RNA-seq in green. **(B)** Expression of *GCLC*, *GSS*, and *GSR* were evaluated with RT-qPCR. Data are presented as median ± standard deviation of n = 5 biologically independent experiments. **(C)** ATAC-seq, ChIP-seq, and RNA-seq read density profiles at the *GCLC, GSS*, and *GSR* loci. **(D)** Monocytes were stimulated with BDG for 24 hours and assessed for total intracellular glutathione levels and GSH/GSSG using calorimetry. Data are presented as median ± standard deviation of n = 5 biologically independent experiments. *P*-values were calculated using paired student’s *t*-test. **P < 0.01; ***P < 0.001.

Given that glutathione metabolism was enriched in the gene ontology of upregulated genes and that glutathione synthesis/metabolism are regulated by NRF2, a TF whose gene expression is increased after BDG exposure and motif is enriched in all the ATAC-seq groups, we sought to investigate the effect of BDG on this pathway in human monocytes (**Figure 4A**). Strikingly, the expression of the genes encoding the enzymes catalyzing GSH synthesis and metabolism *GCLC*, *GSS*, and *GSR* were increased after 24 hours of BDG exposure as assessed by RT-qPCR and RNA-seq (**Figures 4B, C**). At the epigenetic level, 24 hours of BDG exposure was not associated with strong changes in ATAC-seq signal but H3K27ac was increased at the *GCLC* and *GSR* loci. To verify if the increased expression of *GCLC*, *GSS*, and *GSR* had a functional impact on the activity of the GSH pathway, we measured total intracellular glutathione levels and ratio of reduced (GSH) to oxidized (GSSG) glutathione after 24 hours of BDG exposure (**Figure 4D**). In contrast to LPS stimulation, known to increase intracellular ROS and thus increase the intracellular GSH/GSSG ratio without changing total glutathione levels (**Supplementary Figure 3**) (37), BDG exposure increases both the GSH/GSSG ratio and the total amount of intracellular GSH (**Figure 4D**).

### Intracellular GSH Is a Regulator of BDG-Induced NADP Synthesis and Pentose Phosphate Pathway Metabolism

**Figure 5:**
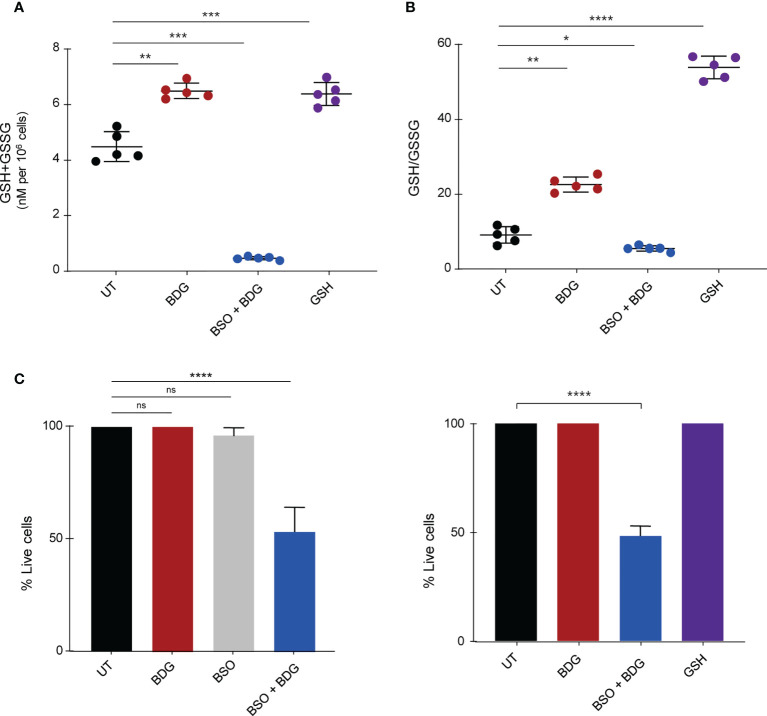
Intracellular glutathione contributes to monocyte survival after BDG exposure. Monocytes were stimulated with BDG for 24 hours, pre-treated with BSO for 24 hours and then stimulated with BDG for 24 hours, or stimulated with GSH for 24 hours and then assessed for **(A)** Intracellular GSH+GSSG (total intracellular glutathione concentration), **(B)** GSH/GSSG (reduced to oxidized glutathione ratio), and **(C)** cell survival. Data are presented as median ± standard deviation of n≥3 biologically independent experiments. *P-*values were calculated using paired one-way ANOVA with multiple comparisons. ns, not significant; *P < 0.05; **P < 0.01; ***P < 0.001; ****P < 0.0001.

To determine the role of the GSH induction by BDG on monocyte function, we evaluated the impact of blocking GSH synthesis using L-buthionine sulfoximine (BSO), an inhibitor of γ-glutamylcysteine synthetase (GCLC) (38). Pretreating culture medium with 120μM of BSO was sufficient to inhibit glutathione synthesis after 48 hours (**Figures 5A, B**). Conversely, as the plasma membrane has transport proteins which can move glutathione between the intra and extracellular environments (39), adding GSH to the culture media increased intracellular levels of GSH (**Figures 5A, B**). Interestingly, while GSH supplementation or BSO pre-treatment alone did not have any effect on monocyte survival, BSO treatment followed by BDG exposure caused significant cell death (**Figure 5C**), indicating a protective effect of the elevated GSH pathway activity during BDG exposure.

**Figure 6:**
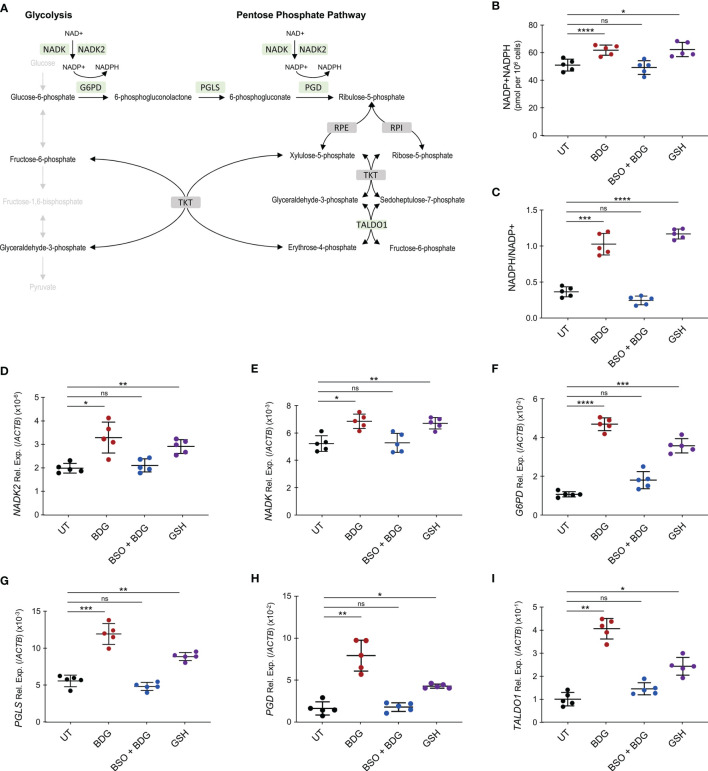
BDG induces NADP synthesis and upregulation of the pentose phosphate pathway (PPP) in a glutathione dependent manner. **(A)** Schema representing PPP with genes that are significantly upregulated after BDG exposure as assessed by RNA-seq in green and genes that are not differentially expressed in grey. **(B–I)** Monocytes were stimulated with BDG for 24 hours, pre-treated with BSO for 24 hours and then stimulated with BDG for 24 hours, or stimulated with GSH for 24 hours and **(B)** total NADP+NADPH levels as well as **(C)** NADPH/NADP+ were measured using calorimetry. The relative expression of **(D)** *NADK*, **(E)** *NADK2*, **(F)** *G6PD*, **(G)** *PGLS*, **(H)** *PGD*, and **(I)** *TALDO1* were quantified using RT-qPCR. Data are presented as median ± standard deviation of n = 5 biologically independent experiments. *P-*values were calculated using paired one-way ANOVA with multiple comparisons. ns, not significant; *P < 0.05; **P < 0.01; ***P < 0.001; ****P < 0.0001.

The pentose phosphate pathway (PPP) was enriched amongst genes significantly upregulated after BDG exposure and one of its products, NADPH, is required for glutathione metabolism (**Figures 6A**). Hence, we sought to investigate the relationship between BDG exposure, glutathione metabolism, and the PPP. Total intracellular levels of NADP+NADPH and NADPH/NADP ratio were increased after BDG exposure (**Figures 6B, C**). Such elevation was abrogated with BSO pre-treatment and replicable with adding GSH to culture media. At the transcriptome level, genes encoding enzymes involved in the pentose phosphate pathway, *NADK*, *NADK2*, *G6PD*, *PGLS*, *PGD*, and *TALDO1* were up-regulated with BDG exposure in a GSH-dependent manner (**Figures 6D**–**I** and **Supplementary Figure 4**). To some extent, glutathione exposure alone was sufficient to induce increased expression of these genes *in vitro*.

### Intracellular GSH Is a Regulator of BDG-Induced NO Secretion, Phagocytosis, and Cytokine Production

**Figure 7:**
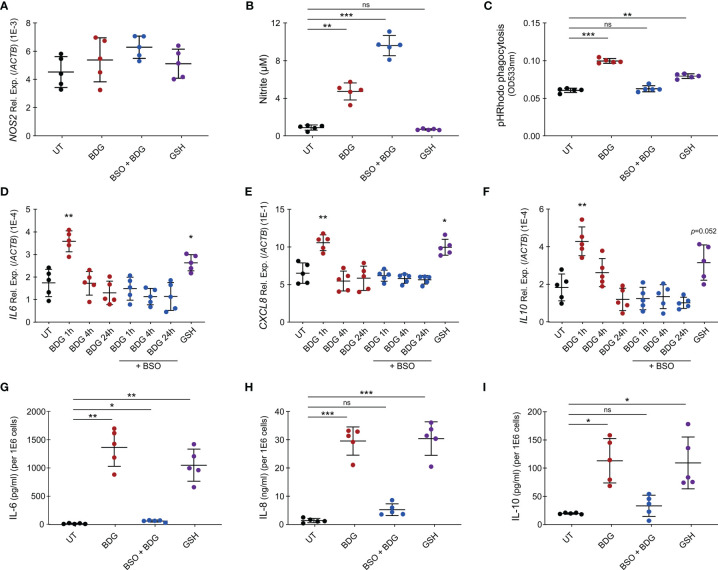
Intracellular glutathione levels modulate monocyte’s inflammatory response to BDG stimulation. **(A–C)** Human monocytes were untreated, stimulated with BDG for 24h, pre-treated with BSO for 24h then stimulated with BDG for 24h, or stimulated with reduced glutathione (GSH) for 24h and assessed for **(A)** *NOS2* expression, **(B)** Nitrite production, and **(C)** phagocytic capacity. **(D–F)** Human monocytes were untreated, stimulated with BDG for 1h, 4h, or 24h, pre-treated with BSO for 24h then stimulated with BDG for 1h, 4h, or 24h, or stimulated with GSH for 24h and then evaluated for expression of the **(D)** *IL-6*, **(E)** *CXCL8*, and **(F)** *IL-10* loci using RT-qPCR. **(G–I)** Human monocytes were untreated, stimulated with BDG for 24h, pre-treated with BSO for 24h then stimulated with BDG for 24h, or stimulated with reduced glutathione (GSH) for 24h and assessed for production of **(G)** IL-6, **(H)** IL-8, **(I)** IL-10 using ELISA. Data are presented as median ± standard deviation of n = 5 biologically independent experiments. *P-*values were calculated using paired one-way ANOVA with multiple comparisons. ns, not significant; *P < 0.05; **P < 0.01; ***P < 0.001.

As metabolism of myeloid cells is closely linked to their effector functions and intracellular GSH levels regulated BDG-induced metabolic changes, we wanted to investigate if GSH could also regulate monocyte’s effector functions in response to BDG. While BDG did not induce increased expression of *NOS2*, we observed that BDG induced production of nitric oxide (NO) (**Figures 7A, B**). Inhibiting glutathione synthesis with BSO increased NO production while exposing monocytes to GSH did not induce any NO. Phagocytic capacity of monocytes was assessed using pH-sensitive rhodopsin labelled *E. coli* that fluoresces in the acidic environment of the phagolysosome. BDG induced increased phagocytic capacity of human monocytes in a GSH-dependent manner (**Figure 7C**). BDG also induced the upregulation of *IL6*, *CXCL8*, and *IL10* gene expression as well as production of IL-6, IL-8, and IL-10 in a GSH-dependent manner, GSH supplementation alone induced a similar response (**Figures 7D**–**I**).

## Discussion

The objective of this study was to better understand the acute inflammatory response of monocytes to the PAMP BDG. Using functional genomics data (ATAC-seq, ChIP-seq, and RNA-seq) (12), we identified that BDG exposure induces changes in the immunometabolic and effector programs of human monocytes. It was previously demonstrated that BDG induces the expression of genes involved in glycolysis, cholesterol synthesis and the pentose phosphate pathways (11) and metabolomics confirmed increased activity of these pathways (40). Herein, we identified that BDG induces glutathione synthesis and metabolism at the epigenetic and transcriptomic levels. *In vitro*, we validated this increased gene expression *via* RT-qPCR and observed that BDG increases the global intracellular concentration of glutathione as well as the level of the oxidized glutathione (GSSG) form. While inhibiting glutathione synthesis with BSO had no effect on survival on its own, BSO pre-treatment followed by BDG exposure had a significant impact on monocyte survival and function. We observed that intracellular glutathione was a regulator of BDG-induced immunometabolic changes by increasing NADP synthesis and the activity of the pentose phosphate pathway. Interestingly, BDG-induced effector functions of monocytes such as NO production, phagocytosis, and cytokine production were also regulated by intracellular levels of glutathione. These results further elucidate the acute response of monocytes to BDG and establish a novel role for glutathione metabolism in monocyte biology.

Monocytes play an important role in clearing infections by detecting PAMPs, phagocytosing/presenting antigens, and producing pro-inflammatory cytokines/chemokines to help recruit other leukocytes to the site of infection. β-glucans are β-D-glucose polysaccharides with different physicochemical properties and found in the cell wall of multiple organisms including fungi, bacteria, yeast, algae, and some cereals. The pathogen-associated β-glucans are insoluble chains of D-glucose linked by 1→3 glycosidic bonds, with some 1→6 branching in yeast and fungi. Recognition of BDG by innate immune cells is required for effective recruitment of other leukocytes and subsequent clearance of the infection (41). Mice deficient for Dectin-1, the myeloid cell receptor for BDG, rendered them susceptible to *Candida* infection. In fact, these mice had substantially increased fungal burdens and enhanced fungal dissemination due to impaired phagocytosis, antigen presentation, and cytokine/ROS production of their monocytes (16). In this study, we have used BDG from the gram negative bacterium *Alcaligenes faecalis* for our *in vitro* experiments while the RNA-seq datasets were generated using heat killed *Candida albicans* (7). Despite their different origin and that fungi BDG harbor 1→6 branching, we believe that these two reagents can be used interchangeably as we have validated the transcriptional regulation of genes induced by fungi BDG using RT-qPCR on monocyte treated with bacterial BDG. Moreover, two recent reports show that BDG from gram negative bacterium (*Alcaligenes faecalis*), zymosan (*fungi*), and yeast-derived BDG induce a similar pro-inflammatory phenotype in primary human monocyte-derived macrophages and primary human monocytes (42, 43). From clinical applications, we know that BDG from *A. faecalis* is extremely similar to fungal BDG, as the clinical BDG test for diagnosing IFIs has false positives in patients with colonization and/or infection with this bacterium (4). Here, while we don’t investigate the precise signaling pathway, we show that intracellular glutathione is required for survival and complete effector function of monocytes in response to BDG. Thus, glutathione may be an important molecule in antifungal immunity and future studies should address its role in clearing fungal infections.

Beyond mycoses, BDG has been shown to induce long-term epigenetic reprogramming, reverse LPS-induced tolerization in monocytes/macrophages, and confer protection from infectious diseases including leishmaniasis and tuberculosis (12–14). Changes in the epigenetic landscape of monocytes leading to BDG-induced trained immunity are mediated by the activation of mechanistic target of rapamycin (mTOR) and hypoxia-inducible factor 1α (HIF-1α). In fact, blocking this pathway in several studies has consistently abrogated BDG-induced trained immunity (11, 44). Interestingly, Mak et al. investigated the molecular targets downstream of intracellular GSH in T cells. As ROS are a known inhibitor of mTOR activation, they showed that intracellular GSH was able to buffer ROS leading to mTOR activation, metabolic reprogramming, and resultant inflammatory response of T cells *in vitro* and *in vivo* (45). In our study, we observed that BDG increased glutathione levels in monocytes and that intracellular GSH was able to reduce ROS production post-translationally. Hence, future studies should investigate whether ROS buffering by intracellular GSH allows BDG-induced trained immunity in monocytes.

Previous studies have also addressed the role of glutathione in monocyte/macrophage biology. Kerstholt et al. demonstrated that *Borrelia burgdorferi* infection of human monocytes increased glutathione synthesis and GSH/GSSG ratio. Moreover, they saw that intracellular glutathione was required for production of acute pro-inflammatory cytokines in response to this bacteria (46). In RAW264.7 macrophages, BSO pre-treatment was previously shown to partially abrogate the LPS-induced pro-inflammatory response (37). Another study investigated the role of glutathione in regulating macrophage mediated killing of *Mycobacterium tuberculosis*. While LPS/IFNγ exposure induced intracellular killing of BCG in J774.1 macrophages, this response was diminished with BSO pre-treatment. To investigate whether this was due to the ability of GSH to buffer ROS, they repeated this experiment with peritoneal macrophages from iNOS knockout mice. Treatment of BCG-infected iNOS^-/-^ macrophages with LPS/IFNγ, induced killing of about 75% of intracellular BCG after 72 hours. In contrast, pre-treating these macrophages with BSO before LPS/IFNγ stimulation completely abrogated this killing and resulted in BCG multiplying after 72 hours (47). Thus, there is evidence that GSH promotes intracellular killing of BCG in a ROS-independent manner. In our study, we observed that intracellular glutathione was a regulator of monocyte’s effector functions including phagocytosis and cytokine production. As previously reported in the RAW264.7 macrophage cell line, we also saw that exogenous GSH was sufficient to elicit cytokine production and increased phagocytic capacity, but the mechanism is poorly understood (48). Taken together, there is converging evidence that suggests a critical role of intracellular glutathione in regulating the acute inflammatory response of monocytes/macrophages.

While there are different models to study glutathione metabolism, in this study we used BSO as it is a specific and potent chemical inhibitor of the rate limiting enzyme GCLC (38). Transient *Gclc* knockdown using siRNA has been successfully use in the mouse hepatocyte cell line FL83B to study the role of glutathione in vitamin D metabolism and other pathways (49, 50). Importantly, the authors have also demonstrated that inhibition of glutathione synthesis using BSO and *Gclc* siRNA knockdown were similarly decreasing cellular GSH levels, increasing oxidative stress and affecting the vitamin D metabolism (50). While future studies can use either *GCLC* knockdown or BSO pretreatment to further investigate the relationship between BDG response and the glutathione pathway, we have chosen chemical inhibition given efficient transient gene knockdown is technically challenging human primary monocytes. Moreover, we have further validated our findings by using exogenous GSH as a positive control. Our *in vitro* experiments on human primary monocytes show that the glutathione metabolism is involved in the regulation of the acute response to BDG at the transcriptomic and molecular levels, yet further studies are required to evaluate the functional impact of this phenomenon *in vivo*. Previous studies have attempted to create *Gclc* knockout mice, however these mice died *in utero* by gestation day 13. Although heterozygous mice were viable and fertile, they only had a 20% reduction in GSH levels making it an unideal model to study glutathione metabolism (51). While *in vivo* models of *Gclc* knockout mice have had limited success, future studies could investigate this relationship with either *Gclc* heterozygous mice or wild type mice treated on the one hand with BSO to pharmacologically block GCLC and on the other hand with exogenous glutathione.

Overall, we investigated the acute response of monocytes to a major PAMP, BDG. We showed that BDG induces glutathione synthesis and metabolism at the epigenetic, transcriptional, and molecular levels. Eliminating intracellular GSH with BSO reduced cell survival in BDG stimulated but not resting monocytes, suggesting a critical role of intracellular GSH in cell survival after an infectious challenge. Moreover, intracellular GSH buffered NO production at the post-translational level and participates in protecting monocytes from their own oxidative stress without abrogating NO production and endosome acidification. BDG was shown to be a regulator of immunometabolism and effector functions of monocytes. We now demonstrate this regulation to be dependent in part on GSH, since BSO pre-treatment abrogated BDG-induced phagocytosis and cytokine production. Overall, our findings demonstrate an important role for GSH in immunity and outline a better understanding of the acute response of monocytes to infections.

## Data Availability Statement

The original contributions presented in the study are included in the article/**Supplementary Material**. Further inquiries can be directed to the corresponding author.

## Ethics Statement

The studies involving human participants were reviewed and approved by McGill University Health Centre (MUHC) Montreal, QC, Canada, REB (#2019-5170). The patients/participants provided their written informed consent to participate in this study.

## Author Contributions

RR and DL designed the study, prepared the first draft of the manuscript, and revised the final draft of the manuscript. RR, MM, JMN, LTT, SI, and JL performed the experiments and analyzed the data. J-PR, AN, and DL supervised the work. All authors contributed to the article and approved the submitted version.

## Funding

This work was supported by a McGill University Faculty of Medicine Startup grant and by a Canada Institute of Health Research (CIHR) Project Grant to DL (#168959) and in part by the Fonds de la Recherche Québec-Santé (FRQ-S): Réseau SIDA/Maladies infectieuses and Thérapie cellulaire. DL was also supported by an FRQ-S, Chercheur-Boursier Junior 1 Award and the Canada Foundation for Innovation John R. Evans Leaders Fund. J-PR is the holder of the Louis Lowenstein Chair in Hematology and Oncology, McGill University. RR was supported by the McGill University J.W. McConnell scholarship and JMA was supported by a McGill University and Consejo Nacional de Ciencia y Tecnología (CONACyT) scholarship. JL is supported by William Turner award holder from the McGill University Health Centre. SI is supported by an award from FRQ-S and CIHR-CTN.

## Conflict of Interest

The authors declare that the research was conducted in the absence of any commercial or financial relationships that could be construed as a potential conflict of interest.

## Publisher’s Note

All claims expressed in this article are solely those of the authors and do not necessarily represent those of their affiliated organizations, or those of the publisher, the editors and the reviewers. Any product that may be evaluated in this article, or claim that may be made by its manufacturer, is not guaranteed or endorsed by the publisher.

## Supplementary Material

Supplementary Figure 1 Gating strategy to validate isolation of human monocytes from PBMC.

Supplementary Figure 2 Longitudinal changes in epigenetic marks in monocytes after BDG exposure. **(A, B)** Clusters of differentially accessible regions identified in **Figure 1A** were queried for **(A)** H3K27ac and **(B)** H3K4me1 ChIP-seq signal in human monocytes after 0h, 1h, 4h, and 24h of BDG exposure. Data are presented as box and whisker plots with box representing median + interquartile range and whiskers representing 5^th^ and 95^th^ percentile marks. Red horizontal line denotes median tag count in 1kb of a specific epigenetic mark at 0h after BDG exposure. *P-*values were calculated using paired one-way ANOVA with multiple comparisons. ns, not significant; **P* < 0.05; ***P* < 0.01; ****P* < 0.001; *****P* < 0.0001.

Supplementary Figure 3 Glutathione synthesis and metabolism in human monocytes before and after 24h stimulation. **(A)** Total intracellular glutathione levels in human monocytes at rest and after 24h of LPS or BDG exposure. **(B)** Intracellular GSH/GSSG ratio in human monocytes at rest and after 24h of LPS or BDG exposure. Data are presented as median ± standard deviation of n=3 biologically independent experiments. *P-*values were calculated using paired one-way ANOVA with multiple comparisons. ns, not significant; **P* < 0.05; ***P* < 0.01; ****P* < 0.001; *****P* < 0.0001.

Supplementary Figure 4 ATAC-seq, ChIP-seq, and RNA-seq read density profiles at the *NADK, NADK2, G6PD, PGLS, PGD*, and *TALDO1* loci.

Supplementary Table 1 Purity of monocyte isolations from PBMC using StemCell CD14+ monocyte negative selection kit.

Supplementary Table 2 List of clusters of differentially accessible regions after BDG exposure and gene ontology enrichment analysis of these clusters.

Supplementary Table 3 Groups of differential gene expression after 1h, 4h, 24h of BDG exposure and gene ontology enrichment analysis of these groups.

Supplementary Table 4 List of primers used for RT-qPCR validations.

Supplementary Table 5 TRUUST analysis of differentially expressed genes after 24 hours of BDG exposure in human monocytes.

## Abbreviations

UT, untreated; BDG, (1→3)-β-D-Glucan; BSO, L-buthionine sulfoximine; GSH, reduced glutathione.
