# Supplementary figures and images for: Glutathione Metabolism Is a Regulator of the Acute Inflammatory Response of Monocytes to (1→3)-β-D-Glucan
Source: Front Immunol. 2021 Nov 11;12:694152. doi: 10.3389/fimmu.2021.694152 (PMC8631827; doi:10.3389/fimmu.2021.694152)

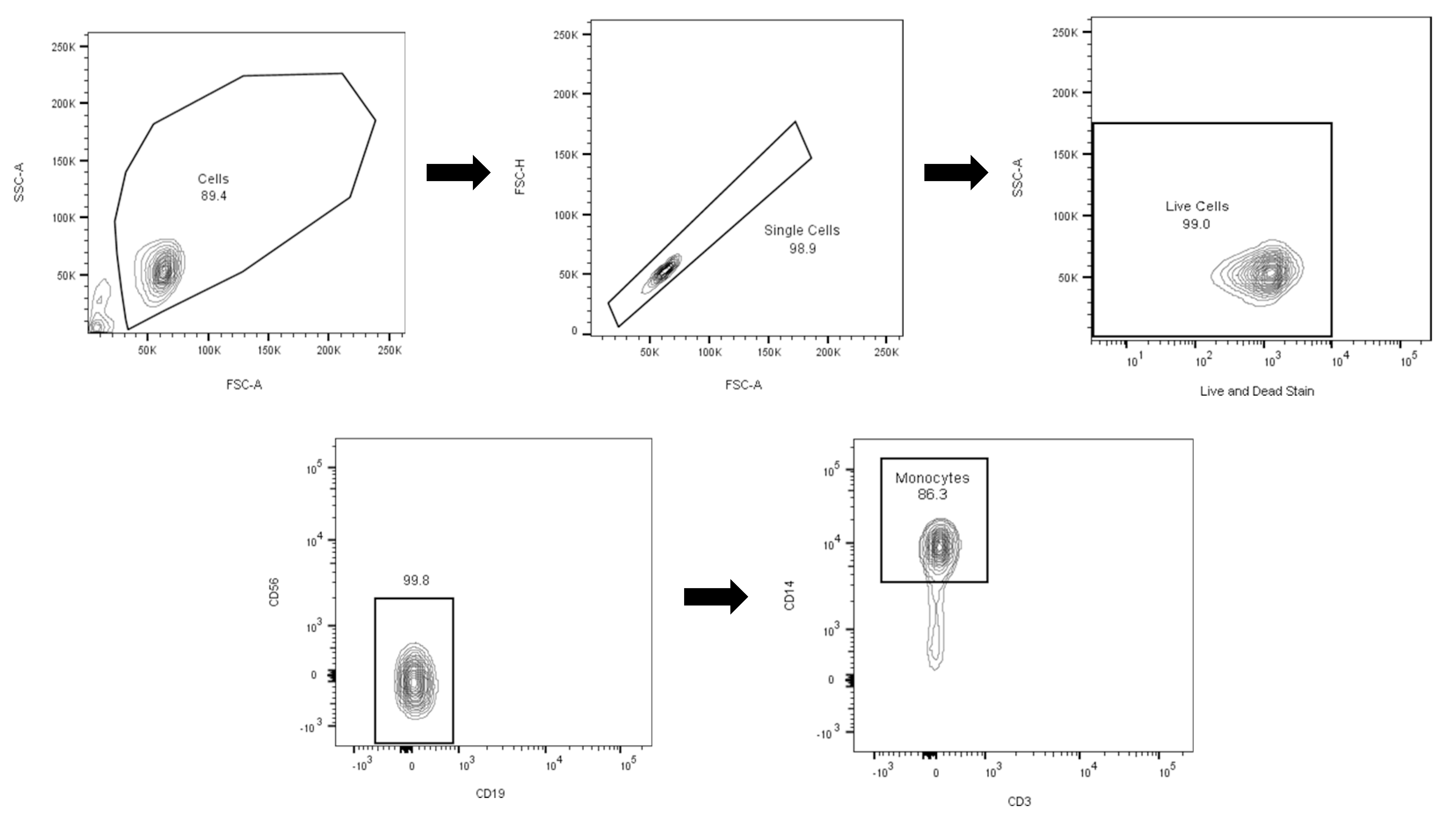

Supplement: Supplementary Figure 1 — Gating strategy to validate isolation of human monocytes from PBMC. [file Image_1.tif]

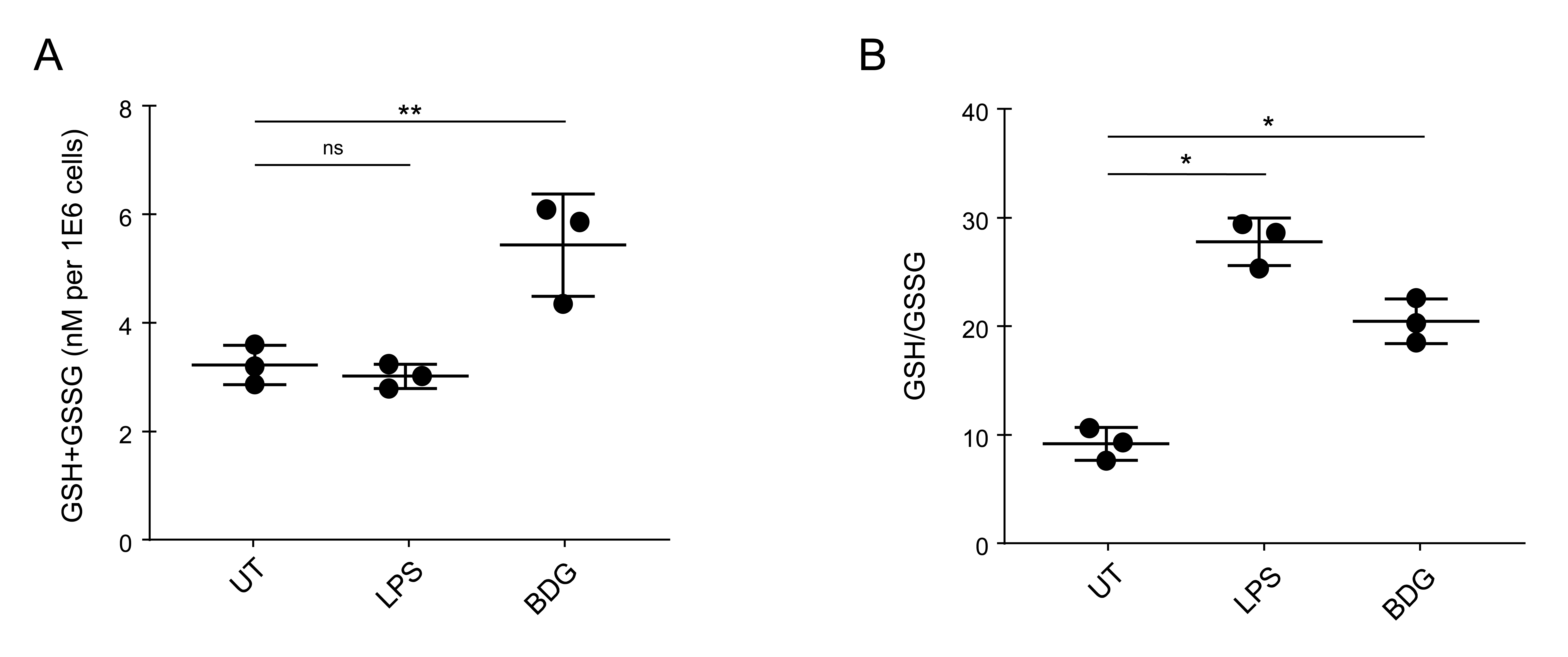

Supplement: Supplementary Figure 3 — Glutathione synthesis and metabolism in human monocytes before and after 24h stimulation. (A) Total intracellular glutathione levels in human monocytes at rest and after 24h of LPS or BDG exposure. (B) Intracellular GSH/GSSG ratio in human monocytes at rest and after 24h of LPS or BDG exposure. Data are presented as median ± standard deviation of n=3 biologically independent experiments. P-values were calculated using paired one-way ANOVA with multiple comparisons. ns, not significant; *P < 0.05; **P < 0.01; ***P < 0.001; ****P < 0.0001. [file Image_3.tif]

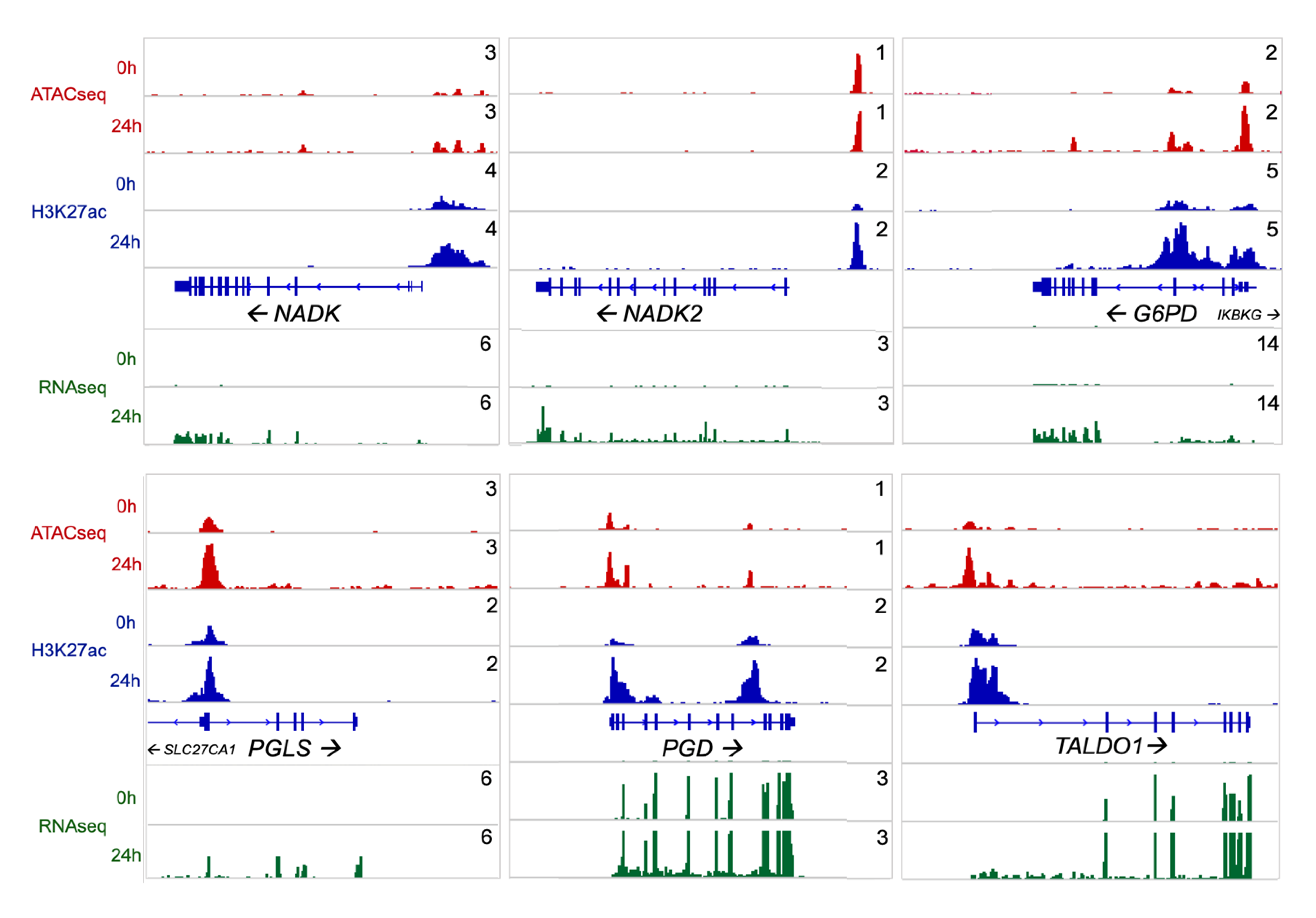

Supplement: Supplementary Figure 4 — ATAC-seq, ChIP-seq, and RNA-seq read density profiles at the NADK, NADK2, G6PD, PGLS, PGD, and TALDO1 loci. [file Image_4.tif]
